# Pulsed Electromagnetic Fields Increased the Anti-Inflammatory Effect of A_2A_ and A_3_ Adenosine Receptors in Human T/C-28a2 Chondrocytes and hFOB 1.19 Osteoblasts

**DOI:** 10.1371/journal.pone.0065561

**Published:** 2013-05-31

**Authors:** Fabrizio Vincenzi, Martina Targa, Carmen Corciulo, Stefania Gessi, Stefania Merighi, Stefania Setti, Ruggero Cadossi, Mary B. Goldring, Pier Andrea Borea, Katia Varani

**Affiliations:** 1 Department of Medical Sciences, Pharmacology Unit, University of Ferrara, Ferrara, Italy; 2 Igea Biophysics Laboratory, Carpi, Italy; 3 Laboratory for Cartilage Biology, Hospital for Special Surgery, Weill Cornell Medical College, New York, New York, United States of America; University of California Merced, United States of America

## Abstract

Adenosine receptors (ARs) have an important role in the regulation of inflammation and their activation is involved in the inhibition of pro-inflammatory cytokine release. The effects of pulsed electromagnetic fields (PEMFs) on inflammation have been reported and we have demonstrated that PEMFs increased A_2A_ and A_3_AR density and functionality in different cell lines. Chondrocytes and osteoblasts are two key cell types in the skeletal system that play important role in cartilage and bone metabolism representing an interesting target to study the effect of PEMFs. The primary aim of the present study was to evaluate if PEMF exposure potentiated the anti-inflammatory effect of A_2A_ and/or A_3_ARs in T/C-28a2 chondrocytes and hFOB 1.19 osteoblasts. Immunofluorescence, mRNA analysis and saturation binding assays revealed that PEMF exposure up-regulated A_2A_ and A_3_AR expression. A_2A_ and A_3_ARs were able to modulate cAMP production and cell proliferation. The activation of A_2A_ and A_3_ARs resulted in the decrease of some of the most relevant pro-inflammatory cytokine release such as interleukin (IL)-6 and IL-8, following the treatment with IL-1β as an inflammatory stimuli. In human chondrocyte and osteoblast cell lines, the inhibitory effect of A_2A_ and A_3_AR stimulation on the release of prostaglandin E_2_ (PGE_2_), an important lipid inflammatory mediator, was observed. In addition, in T/C-28a2 cells, the activation of A_2A_ or A_3_ARs elicited an inhibition of vascular endothelial growth factor (VEGF) secretion. In hFOB 1.19 osteoblasts, PEMF exposure determined an increase of osteoprotegerin (OPG) production. The effect of the A_2A_ or A_3_AR agonists in the examined cells was enhanced in the presence of PEMFs and completely blocked by using well-known selective antagonists. These results demonstrated that PEMF exposure significantly increase the anti-inflammatory effect of A_2A_ or A_3_ARs suggesting their potential therapeutic use in the therapy of inflammatory bone and joint disorders.

## Introduction

Chronic inflammation represent an important factor in the pathophysiology of several joint diseases [1]. In human joint damages, chondrocytes are able to respond to the depletion of extracellular matrix and abnormal biomechanical functions trying to preserve matrix integrity [2]. The degradation of the cartilage matrix is mediated by a number of different factors including pro-inflammatory cytokines, matrix degrading enzymes, nitric oxide (NO), oxygen derived free radicals and prostaglandins [3], [4].

On the other hand continuous remodeling by bone cells such as osteoblasts and osteoclasts allows the skeleton to grow, adapt and repair itself [5], [6]. In healthy adults bone remodeling, an important homeostatic function, is well balanced and abnormalities in this process can result in a variety of skeletal disorders [7]. The number of osteoblasts decreases with age, affecting the balance of formation and resorption in the bone tissue, and potentially leading to osteoporosis [8]. Moreover, osteoblasts if adequately stimulated produce increased levels of pro-inflammatory cytokines [9]. It is well accepted that osteoblasts release the receptor activator of nuclear factor kB (NF-kB) ligand (RANKL) modulating signaling pathways that promote osteoclast differentiation and survival [10]. Moreover, osteoblasts also produce a protein named osteoprotegerin (OPG) that, preventing the biological effects of RANKL, plays an important osteoprotective role [11].

Previous papers have reported that PEMF exposure could act modulating cartilage and bone metabolism, stimulating chondrocyte and/or osteoblast cell proliferation and the synthesis of extracellular matrix components [12]. The stimulation of chondrocyte and/or osteoblast cell proliferation induced by PEMFs has been shown to have a positive effect in the treatment of fracture healing [13], [14]. In particular, a well observed beneficial effect on osteogenesis has been reported based on the observation that PEMFs stimulate cell proliferation, induce osteoblastogenesis and differentiation of osteoblasts [15]. In addition, PEMFs stimulate proteoglycan synthesis without affecting the degradation suggesting their potential use to preserve cartilage integrity and function [16]. A clinical study has shown that PEMF treatment after arthroscopic surgery results in faster and complete functional recovery compared to controls in the short term, that is maintained at 3 year follow-up [17].

Adenosine, an endogenous modulator of a wide range of biological functions, interacts with four cell surface subtypes classified as A_1_, A_2A_, A_2B_ and A_3_ adenosine receptors (ARs) [18]. A_1_ and A_3_ARs inhibit adenylate cyclase activity and decrease cAMP production whilst A_2A_ and A_2B_ARs exert an increase of cAMP accumulation [19]. Modulation of ARs has an important role in the regulation of inflammatory processes suggesting their involvement in different pathologies based on inflammation [20]–[23]. Recently, it has been well documented that the adenosine and its receptors are involved in bone remodeling. Adenosine A_1_AR-knockout mice are protected from bone loss suggesting that these receptor subtypes may be a useful target in treating diseases characterized by excessive bone turnover [24], [25]. Other studies reported that A_2B_ or A_2A_AR stimulation could be implicated in osteoblastic differentiation revealing their involvement in bone formation and fracture repair [26], [27].

It has been reported that different physiological systems seem to be influenced by PEMF exposure as revealed by *in vitro* experiments. The effect of PEMFs on ARs in various cells and tissues such as in human neutrophils has been investigated [28], [29]. The treatment with PEMFs induced a selective increase in A_2A_ARs expressed in rat cortex membranes and in rat cortical neurons [30]. A potentiated anti-tumoral effect of A_3_ARs by PEMFs was found in different cell lines such as rat adrenal pheochromocytoma (PC12) and human glioblastoma (U87MG) cell lines [31]. A role of ARs and PEMFs in modulating bovine chondrocytes and synoviocytes activity has been previously documented [32], [33]. Functional studies in human synoviocytes have suggested an anti-inflammatory effect linked to A_2A_ARs that is primarily based on the inhibition of PGE_2_ production [34]. No papers are present in literature studying the effect of PEMFs and ARs in human chondrocytes and osteoblasts despite their co-presence could be very interesting in the cell functionality.

In the present study we have investigated whether PEMFs modulate the expression of ARs in T/C-28a2 chondrocytes and in hFOB 1.19 osteoblasts. The effect of one of the most important pro-inflammatory stimuli such as IL-1β on A_2A_ and A_3_ARs in the absence or in the presence of PEMFs has been examined. The stimulation of A_2A_ or A_3_ARs has been investigated on cAMP production and cell proliferation as well as on the release of PGE_2_ and some of the most relevant pro-inflammatory cytokines such as IL-6 or IL-8. Moreover, the effects of A_2A_ or A_3_AR agonists on vascular endothelial growth factor (VEGF) release in T/C-28a2 chondrocytes and on OPG production in hFOB 1.19 osteoblasts have been explored. In addition, the activation of A_2A_ or A_3_ARs has been examined on NF-kB activation. All the experiments were carried out in the absence or in the presence of PEMF exposure. These results could indicate the possibility of novel therapeutic approaches based on the interaction of AR modulation and a non-invasive stimulus represented by PEMFs.

## Materials and Methods

### Cell culture

Human immortalized chondrocyte cells (T/C-28a2) were kindly provided by Professor Mary B. Goldring, from Cornell Medical College, NY, USA [35], [36]. Cells were cultured in complete medium DMEM F12 (1∶1), containing 10% FBS. Cells were grown at 37°C, in 5% CO2. T/C-28a2 cells represent an appropriate chondrocyte model as suggested by the significant similarities between human primary chondrocytes and T/C-28a2 cells in the induction of IL-6 synthesis in response to chemical and shear stimulation [37].

Human fetal osteoblast cells (hFOB 1.19) were obtained from ATCC (Manassas, VA, USA). Cells were cultured in Ham's F12 without phenol red (Gibco, Invitrogen, Carlsbad, CA), containing 10% fetal bovine serum, 0.3 mg/ml G418. Cells were grown at a permissive temperature of 34°C for a rapid cell division and the medium was renewed every 2 to 3 days [38].

### Field Exposure System

T/C-28a2 chondrocytes or hFOB 1.19 osteoblasts were exposed to PEMFs generated by a pair of rectangular horizontal coils (14 cm×23 cm), each made of 1400 turns of copper wire placed opposite to each other. The culture was placed between this pair of coils so that the plane of the coils was perpendicular to the culture flasks. The coils were powered by the PEMF generator system (IGEA, Carpi, Italy) used in previous studies [30]–[32], which produced a pulsed signal with the following parameters: pulse duration of 1.3 ms and frequency of 75 Hz.

The peak intensity of the magnetic field was 1.5±0.1 mT for T/C-28a2 chondrocytes or 2.5±0.2 mT for hFOB 1.19 osteoblasts. It was detected in air between two coils from one side to the other, at the level of the culture flasks, using the Hall probe (HTD61-0608-05-T, F.W. Bell, Sypris Solutions, Louisville, KY, USA) of a gaussmeter (DG500, Laboratorio Elettrofisico, Milan, Italy) with a reading sensitivity of 0.2%. The peak values measured between two coils in air had a maximum variation of 1% in the whole area in which the culture flasks were placed.

### RT-PCR analysis of ARs

Total cytoplasmic RNA was extracted by the acid guanidiniumthiocyanate-phenol method from T/C-28a2 chondrocytes or hFOB 1.19 osteoblasts untreated or treated with PEMFs for 24 hours. Quantitative real-time RT-PCR assay of mRNAs was carried out using a gene-specific, fluorescently labeled TaqMan minor groove binder (MGB) probe in an ABI Prism 7700 Sequence Detection System (Applied Biosystems, Warrington Cheshire, UK). For the real-time RT-PCR of A_1_, A_2A_, A_2B_ and A_3_ARs the Assays-on-Demand gene expression products (Applied Biosystems) were used. For the real-time RT-PCR of the reference gene, the endogenous control human glyceraldehyde-3-phosphate dehydrogenase (GAPDH) kit was used, and the probe was labeled with the fluorescent dye VIC (Applied Biosystems). In the negative control, sterile distilled water was added instead of template [39].

### Western blot assay for ARs

T/C-28a2 chondrocytes or hFOB 1.19 osteoblasts untreated or treated with PEMFs for 24 hours were lysed in Triton lysis buffer and aliquots of total protein samples (50 µg) were analysed using specific A_1_, A_2A_, A_2B_ and A_3_AR antibody (Alpha Diagnostic, San Antonio, TX, USA). Filters were washed and incubated for 1 hour at room temperature with peroxidase-conjugated secondary antibody (1∶2000 dilution). Specific reaction were revealed with enhanced chemiluminescence Western blotting detection reagent (GE Healthcare). Western blotting assays were also normalized against the housekeeping protein β-actin [20].

### Saturation binding experiments to ARs

Saturation binding experiments to A_1_ARs were performed according to the method described previously using [^3^H]-1,3-dipropyl-8-cyclopentyl-xanthine ([^3^H]-DPCPX, specific activity 120 Ci/mmol; Perkin-Elmer, Boston, MA, USA) as radioligand. The membranes derived from PEMFs-treated or untreated T/C-28a2 or hFOB 1.19 cells (100 µg of protein/assay) with different concentrations of the radioligand [^3^H]-DPCPX (0.1–30 nM) were incubated in Tris–HCl 50 mM, pH 7.4, for 90 min at 4°C. Nonspecific binding was determined in the presence of DPCPX 1 µM [40]. Saturation binding experiments to A_2A_ARs were performed according to the method described previously using [^3^H]-4-(2-[7-amino-2-(2-furyl)[1,2,4] triazolo [2,3-a] [1,3,5] triazin-5-yl-amino]ethyl ([^3^H]-ZM 241385, specific activity 27.4 Ci/mmol; American Radiolabeled Chemicals Inc, Saint Louis, MO, USA) as radioligand. The membranes derived from PEMFs-treated or untreated T/C-28a2 or hFOB 1.19 cells (100 µg of protein/assay) were incubated for 60 min at 4°C with various concentrations of the radioligand [^3^H]-ZM 241385 (0.1–30 nM) and Tris–HCl 50 mM, MgCl_2_ 10 mM, pH 7.4. Nonspecific binding was determined in the presence of ZM 241385 1 µM [39]. Saturation binding experiments to A_2B_ARs were performed using [^3^H]-N-benzo [1,3[dioxol-5-yl-2-[5-(2,6-dioxo-1,3-dipropyl-2,3,6,7-tetrahydro-1H-purin-8-yl)-1-methyl-1H-pyrazol-3-yl-oxy]-acetamide ([^3^H]-MRE 2029F20, specific activity 123 Ci/mmol; GE Healthcare, Little Chalfont, UK) as radioligand. The membranes obtained as previously described (100 µg of protein/assay) with [^3^H]-MRE 2029F20 in the range 0.1–30 nM were incubated in Tris–HCl 50 mM, MgCl_2_ 10 mM, EDTA 1 mM, pH 7.4 at 4°C for 60 min. Nonspecific binding was determined in the presence of MRE 2029F20 1 µM [41]. Saturation binding experiments to A_3_ARs were performed using [^3^H]-5N-(4-methoxyphenylcarbamoyl) amino-8-propyl-2-(2-furyl) pyrazolo [4,3-e]-1,2,4-triazolo [1,5-c]pyrimidine ([^3^H]-MRE 3008F20, specific activity 67 Ci/mmol; GE Healthcare) as radioligand. The membranes treated as above mentioned (100 µg of protein/assay) with [^3^H]-MRE 3008F20 (0.1–50 nM) were incubated in Tris–HCl 50 mM, MgCl_2_ 10 mM, EDTA 1 mM, pH 7.4, at 4°C for 150 min. Nonspecific binding was determined in the presence of MRE 3008F20 1 µM [42]. At the end of the incubation time, bound and free radioactivity was separated by filtering the assay mixture through Whatman GF/B glass fiber filters by using a Brandel cell harvester. The filter bound radioactivity was counted by Scintillation Counter Perkin Elmer Tri Carb 2810 TR with an efficiency of 62% (Perkin-Elmer).

To verify the effect of pro-inflammatory stimuli on ARs, T/C-28a2 chondrocytes or hFOB 1.19 osteoblasts were also treated with IL-1β at 1 ng/ml for 48 hours, in the absence or in the presence of PEMFs for the last 24 hours. At the end of the treatment the cells were used in saturation binding experiments for A_2A_ or A_3_ARs or in functional experiments.

### Immunoflourescence staining of A_2A_ and A_3_ARs

T/C-28a2 chondrocyte or hFOB 1.19 osteoblast cells grown on coverslips and incubated in polylysine-treated chambers were fixed with 4% formalin in PBS, pH 7.4 for 15 min at room temperature. After two or five min washes with ice cold PBS, potential sites for nonspecific antibody binding were blocked by 30 min incubation with 1% BSA in PBST pH 7.4. The cells were then incubated with specific A_2A_ or A_3_ARs polyclonal primary antibody (1∶50 dilution) overnight at 4°C (Alpha Diagnostics Inc). Subsequently, they were incubated with secondary antibody (1∶80) conjugated to fluorescein isothiocyanate (FITC) goat anti-rabbit IgG for 1 hour at room temperature and nuclear stain with 4′,6-diamidino-2- henylindole (DAPI, Sigma) 1 µg/ml for 20 min. After washing with PBS, pH 7.4, the cells were mounted for microscopy with DABCO (1,4-Diazabicyclo (2.2.2) octane, Sigma) and visualized by a microscopy Nikon Eclipse 50i [43].

### Measurement of cyclic AMP levels

T/C-28a2 chondrocytes or hFOB 1.19 osteoblasts (10^6^ cells per sample) were suspended in 0.5 ml incubation mixture Krebs Ringer phosphate buffer, containing 1.0 IU/ml adenosine deaminase (Sigma) and preincubated for 10 min in a shaking bath at 37°C with 0.5 mM of 4-(3-butoxy-4-methoxybenzyl)-2-imidazolidinone (Ro 20-1724) as phosphodiesterase inhibitor. Then the effects of the A_2A_AR agonist 2-*p*-(2-carboxyethyl)phenethylamino-5′-N-ethylcarboxamidoadenosine (CGS 21680, Sigma) or of the A_3_AR agonist 2-chloro-*N* ^6^-(3-iodobenzyl) adenosine-5′-*N*-methyl-uronamide (Cl-IB-MECA, Tocris, Bristol, UK) at 100 nM concentration were studied. To better investigate the inhibitory effect of Cl-IB-MECA, the cells were also incubated with forskolin (1 µM). A_2A_ or A_3_ARs selected adenosine antagonists, such as 2-(2-Furanyl)-7-[3-(4-methoxyphenyl)propyl]-7H-pyrazolo [4,3-e][1,2,4]triazolo[1,5-c]pyrimidin-5-amine (SCH 442416, Sigma) or 1,4-dihydro-2-methyl-6-phenyl-4-(phenylethynyl)-3,5-pyridinedicarboxylic acid 3-ethyl-5-[(3-nitrophenyl)methyl] ester (MRS 1334, Tocris) at the 1 µM concentration, were also used to verify the specific involvement of these subtypes in cAMP production. The final aqueous solution was tested to evaluate cAMP levels by using a competition binding protein assay with [^3^H]-cAMP, trizma base 0.1 mM, aminophylline 8.0 mM, mercaptoethanol 6.0 mM, pH 7.4 [39]. At the end of the incubation time (150 min at 4°C), and after the addition of charcoal, the samples were centrifuged at 2000× g for 10 min and the clear supernatant was counted in a liquid Scintillation Counter Tri Carb Perkin-Elmer 2810 TR.

### Cell Proliferation Assays

T/C-28a2 or hFOB 1.19 cells were seeded in fresh medium with 1 µCi/ml [^3^H]-Thymidine for 24 hours and simultaneously treated with well-known adenosine agonists such as CGS 21680 or Cl-IB-MECA (100 nM) in the absence or presence of SCH 442416 or MRS 1334 (1 µM). Proliferation assays under the same experimental conditions were also carried out in the presence of PEMF exposure. After 24 hours of labeling, cells were trypsinized, dispensed in four wells of a 96-well plate, and filtered through Whatman GF/C glass fiber filters using a Micro-Mate 196 cell harvester (Perkin-Elmer). The filter-bound radioactivity was counted on Top Count Microplate Scintillation Counter with Micro Scint 20 [43].

### IL-6, IL-8, PGE_2_, VEGF and OPG release

T/C-28a2 chondrocytes or hFOB 1.19 osteoblasts were seeded into 24-well plates and incubated in the absence or in the presence of IL-1β (1 ng/ml) for 48 hours. Some of the cells were also incubated in the absence or in the presence of CGS 21680 or Cl-IB-MECA (1 µM). Selective A_2A_ or A_3_AR antagonists such as SCH 442416 or MRS 1334 (1 µM) were used to verify the specific involvement of these receptors in cytokine release. In order to examine the effect of PEMFs the cells were also treated for the last 24 hours in comparison with untreated cells. At the end of incubation, the cell suspension was collected and centrifuged at 1000× g for 10 min at 4°C. The pro-inflammatory cytokines IL-6 and IL-8, the lipid mediator PGE_2_ and the angiogenic factor VEGF levels were determined with specific quantitative sandwich ELISA kit (R&D Systems, Minneapolis, MN, USA) according to the manufacturer instructions [33], [39], [44]. The production of OPG from hFOB 1.19 osteoblasts was determined by ELISA kit (Abcam, Cambridge, UK) following the manufacturer instructions. Briefly, the reaction was developed with streptavidin-horseradish peroxidase and optical density was read at 450 nm wavelength.

### NF-kB activation

Nuclear extracts from T/C-28a2 chondrocytes or hFOB 1.19 osteoblasts were obtained by using a nuclear extract kit (Active Motif, Carlsbad, CA, USA) according to the manufacturer instructions. The NF-kB activation was evaluated by detecting phosphorylated p65 proteins in nuclear extracts by using the TransAM NF-kB kit (Active Motif, Carlsbad, USA). Phosphorylated NF-kB subunits specifically binds to the immobilized oligonucleotides containing the NF-kB consensus site (5′-GGGACTTTCC-3′). The primary antibody used to detect NF-kB recognized an epitope in the subunits that is accessible only when it is activated and bound to its DNA target. A horseradish peroxidase-conjugated secondary antibody provided a sensitive colorimetric readout that was quantified by spectrophotometry at 450 nm wavelength [21].

### Statistical Analysis

Dissociation equilibrium constants for saturation binding, affinity or K_D_ values, as well as the maximum densities of specific binding sites, Bmax were calculated for a system of one or two-binding site populations by non-linear curve fitting using the program Ligand purchased from Kell Biosoft, Ferguson, MO, USA [31], [39]. All data are reported as mean ± SEM of different independent experiments as indicated in Result section or in Figure legend. Analysis of data was performed by one-way analysis of variance (ANOVA) followed by Dunnett's test or unpaired two-sided Student's t-test for comparison of two samples and were considered statistically significant with a p value less than 0.05 (Graph Pad Software, San Diego, CA, USA).

## Results

### mRNA and protein expression of ARs in T/C-28a2 and hFOB 1.19 cells

**Figure 1:**
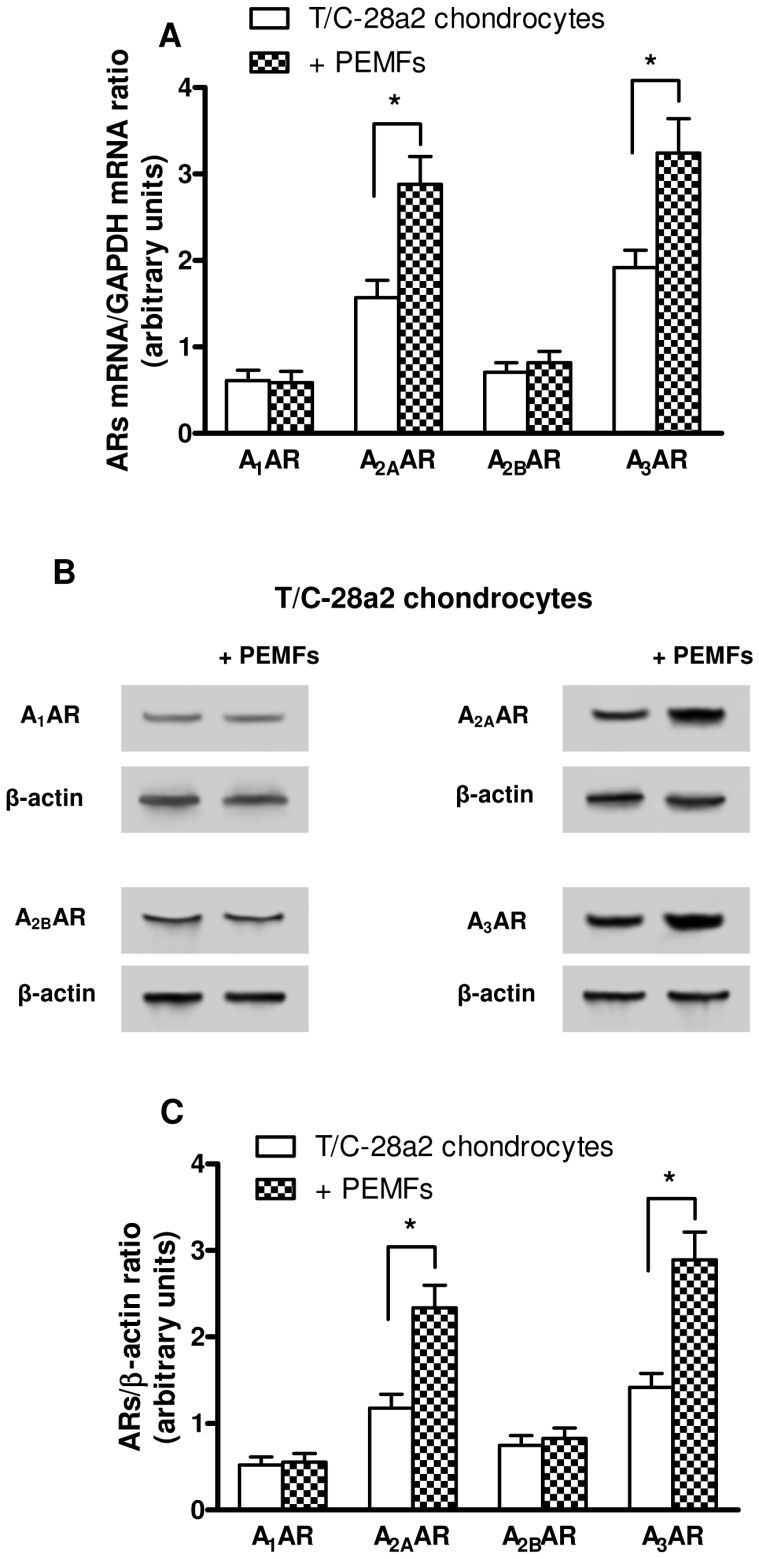
mRNA levels and protein expression of ARs in T/C-28a2 chondrocytes. (A) Relative A_1_, A_2A_, A_2B_ and A_3_AR mRNA levels in T/C-28a2 cells untreated or treated with PEMFs for 24 hours, normalized by using GAPDH mRNA as internal control. (B) Representative Western blotting analysis showing the immunoblot signals of A_1_, A_2A_, A_2B_ and A_3_ARs in T/C-28a2 chondrocytes treated or untreated with PEMFs for 24 hours. (C) Densitometric analysis showing the ratio between ARs and β-actin. Data are reported as the mean ± SEM of six independent experiments. *, p<0.01.

Figure 1A shows the relative mRNA levels of A_1_, A_2A_, A_2B_ and A_3_ARs in T/C-28a2 chondrocytes obtained by real-time quantitative RT-PCR. The treatment of the cells with PEMFs for 24 hours elicited a statistical significant increase of A_2A_ and A_3_ARs mRNA levels (p<0.01 vs control conditions). The upregulation of A_2A_ and A_3_ARs protein expression in T/C-28a2 chondrocytes following PEMF exposure was confirmed by Western blot assays (Figure 1B). In particular, densitometric analysis revealed an increase of 2.1 fold and 2.3 fold for A_2A_ and A_3_ARs, respectively (Figure 1C, p<0.01 vs control conditions). PEMF treatment did not determined any changes in mRNA or protein expression for A_1_ and A_2B_ARs in T/C-28a2 cells (Figure 1).

**Figure 2:**
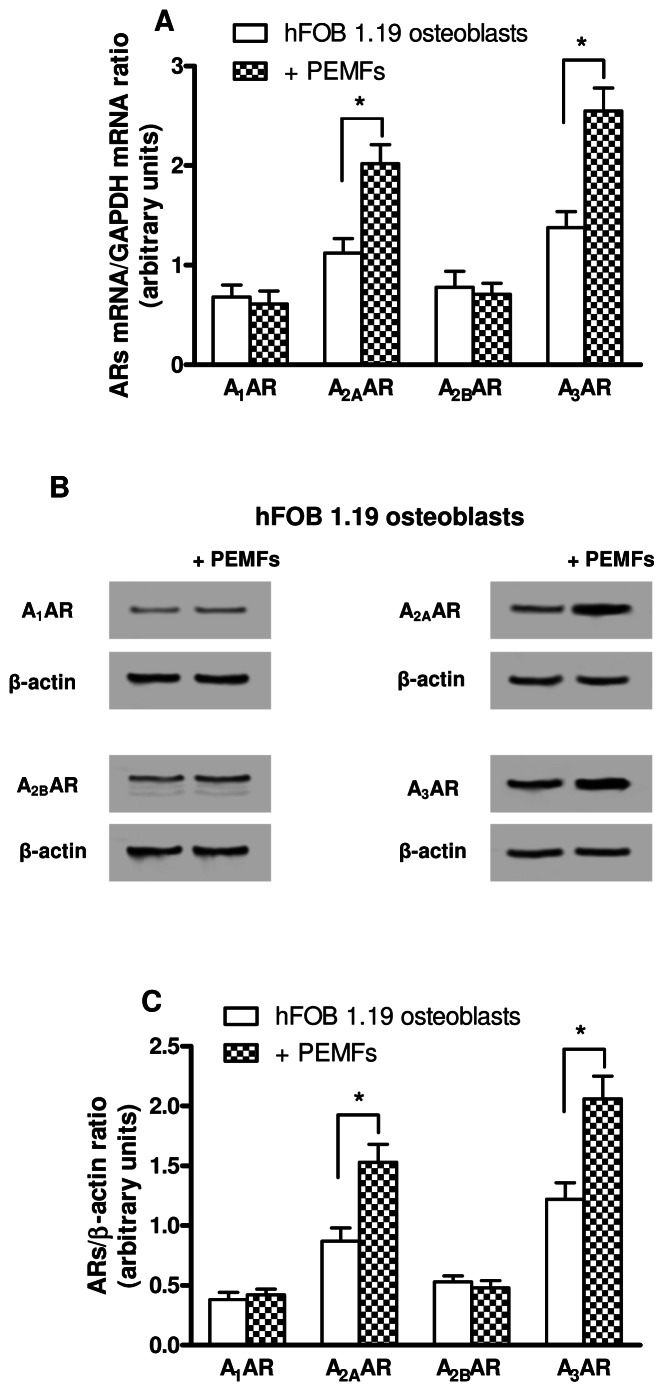
mRNA levels and protein expression of ARs in hFOB 1.19 osteoblasts. (A) Relative A_1_, A_2A_, A_2B_ and A_3_AR mRNA levels in hFOB 1.19 cells untreated or treated with PEMFs for 24 hours, normalized by using GAPDH mRNA as internal control. (B) Representative Western blotting analysis showing the immunoblot signals of A_1_, A_2A_, A_2B_ and A_3_ARs in hFOB 1.19 osteoblasts treated or untreated with PEMFs for 24 hours. (C) Densitometric analysis showing the ratio between ARs and β-actin. Data are reported as the mean ± SEM of six independent experiments. *, p<0.01.

Analogous results were obtained in hFOB 1.19 cells following PEMF treatment (Figure 2). A_2A_ and A_3_AR mRNA levels (Figure 2A) as well as their protein expression (Figure 2B and C) were augmented by PEMFs while A_1_ and A_2B_ARs were not affected by PEMF exposure.

### ARs saturation binding experiments in T/C-28a2 and hFOB 1.19 cells

**Figure 3:**
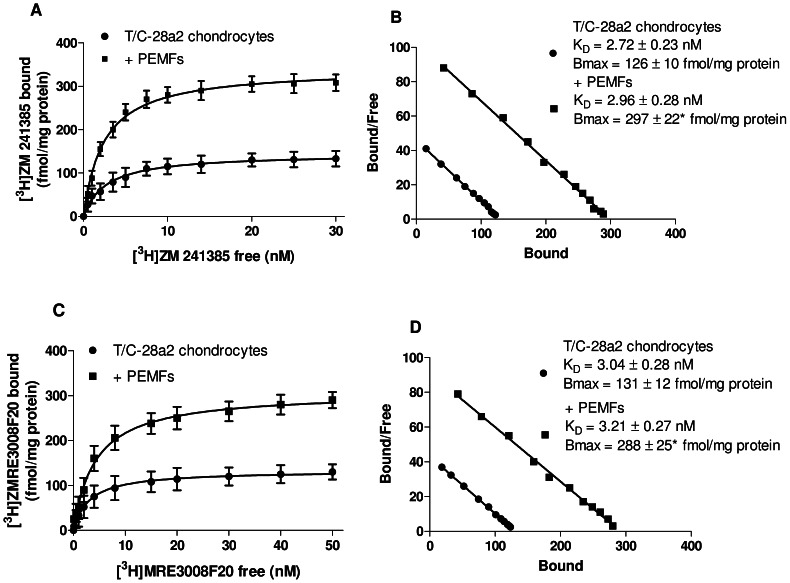
PEMF exposure up-regulated A_2A_ and A_3_ARs in T/C-28a2 chondrocytes. Saturation curves and Scatchard plots of [^3^H] ZM 241385 binding to A_2A_ARs (A, B) and of [^3^H] MRE 3008F20 binding to A_3_ARs (C, D) on membranes from T/C-28a2 chondrocyte cells before and after 24 hours of PEMF exposure. Results are reported as the mean ± SEM of six independent experiments. *, p<0.01 vs untreated T/C-28a2 chondrocytes.

Saturation binding experiments were performed to evaluate the affinity (K_D_) and density (Bmax) of ARs in T/C-28a2 and hFOB 1.19 cells and to better quantify the upregulation of A_2A_ and A_3_ARs determined by PEMF exposure. Table 1 shows that the K_D_ and Bmax values for A_1_ and A_2B_ARs were not affected by PEMF exposure, while it determined an increase of A_2A_ and A_3_AR density in both the cell line examined. In T/C-28a2 chondrocytes the affinity of the radioligand [^3^H]-ZM241385 for A_2A_ARs did not change after 24 hours PEMF treatment whereas the Bmax value increased from 126±10 to 297±22* fmol/mg protein (*, p<0.01, Figure 3A, B). In the same cells, saturation binding experiments with the radioligand [^3^H]-MRE3008F20 revealed a 2.2 fold of increase in A_3_AR density following PEMF treatment (Figure 3C, D). For A_2A_ and A_3_ARs, the linearity of the Scatchard plots (Figure 3B and D, respectively) indicated the presence of a single class of high affinity binding site.

**Table 1:** Affinity and density of A_1_, A_2A_, A_2B_ and A_3_ARs in untreated or PEMF treated T/C-28a2 chondrocytes or hFOB 1.19 osteoblasts.

	A_1_ARs		A_2A_ARs		A_2B_ARs		A_3_ARs	
	K_D_	Bmax	K_D_	Bmax	K_D_	Bmax	K_D_	Bmax
**T/C-28a2**	2.49±0.23	27±2	2.72±0.23	126±10	2.67±0.28	58±5	3.04±0.28	131±12
**+PEMFs**	2.37±0.22	28±3	2.96±0.28	297±22*	2.85±0.26	55±6	3.21±0.27	288±25*
**hFOB 1.19**	1.21±0.11	26±2	2.52±0.21	110±11	1.75±0.15	38±4	3.02±0.26	135±12
**+PEMFs**	1.27±0.10	29±3	2.47±0.22	275±23*	1.82±0.16	41±4	3.21±0.27	328±29*

Affinity and density are expressed as K_D_, nM and Bmax, fmol/mg protein, respectively. Data are expressed as mean (n = 6) ± SEM.

* , p<0.01 vs the examined cells in the absence of PEMFs.

**Figure 4:**
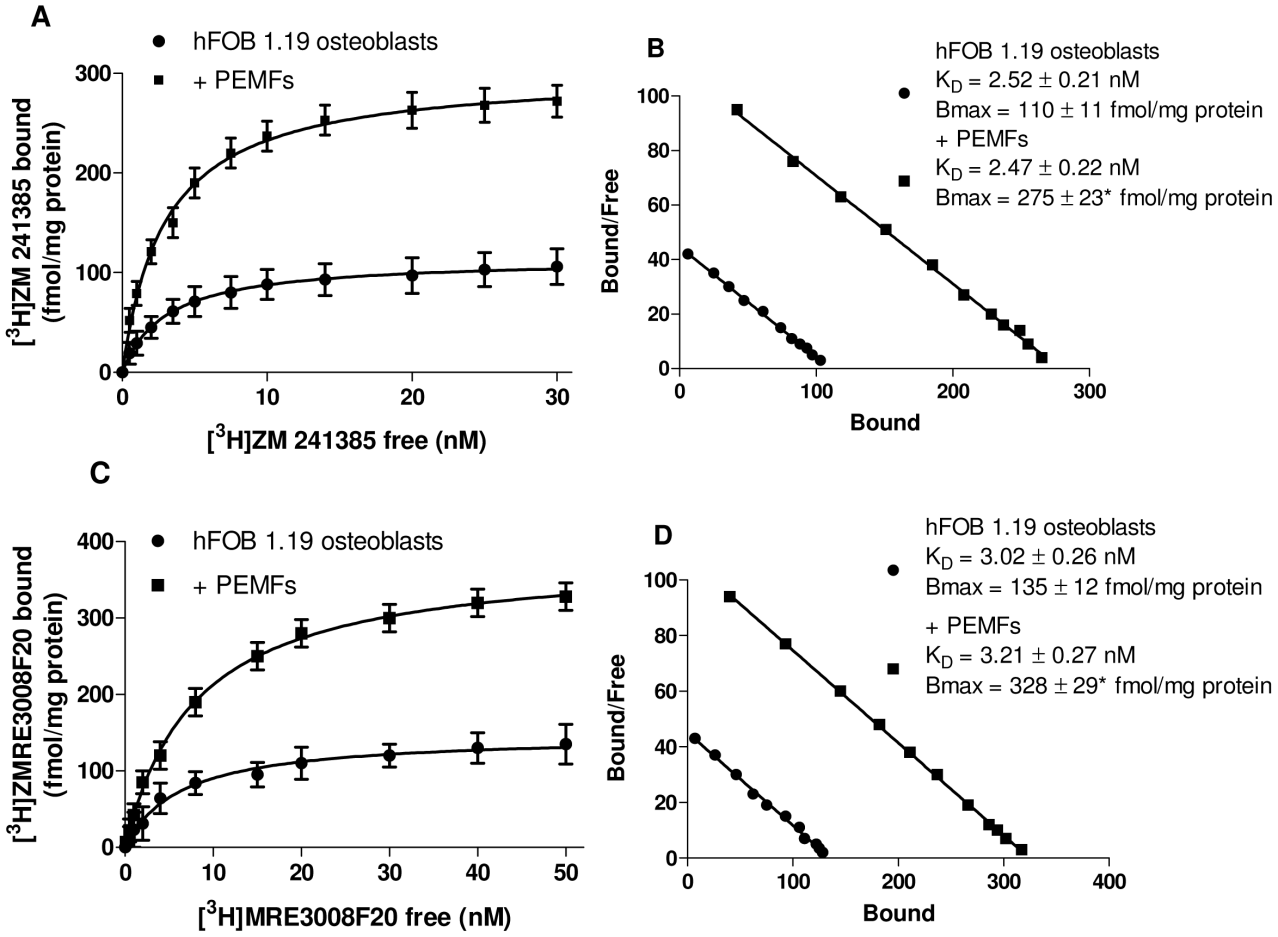
PEMF exposure up-regulated A_2A_ and A_3_ARs in hFOB 1.19 osteoblasts. Saturation curves and Scatchard plots of [^3^H] ZM 241385 binding to A_2A_ARs (A, B) and of [^3^H] MRE 3008F20 binding to A_3_ARs (C, D) on membranes from hFOB 1.19 osteoblast before and after 24 hours of PEMF exposure. Results are reported as the mean ± SEM of six independent experiments. *, p<0.01 vs untreated hFOB 1.19 osteoblasts.

In hFOB 1.19 cells PEMF exposure determined an increase from 110±11 to 275±23* fmol/mg protein and from 135±12 fmol/mg protein to 328±29* fmol/mg protein for A_2A_ and A_3_AR Bmax values, respectively (*, p<0.01, Figure 4). PEMF exposure did not induced any changes in the affinity values for the A_2A_ and A_3_ARs in hFOB 1.19 cells. In particular, the K_D_ values of the radioligand [^3^H]-ZM241385 for A_2A_ARs were 2.52±0.21 nM and 2.47±0.22 nM in the absence or in the presence of PEMFs, respectively (Figure 4A, B). Similarly, the K_D_ values of the radioligand [^3^H]-MRE3008F20 for A_3_ARs were 3.02±0.26 nM and 3.21±0.27 nM in the absence or in the presence of PEMFs, respectively (Figure 4C, D). These data suggested that PEMF exposure did not influenced the ligand-receptor interaction but increased the receptor expression in the membrane surface.

Since most of the functional experiments were performed in the presence of the pro-inflammatory cytokine IL-1β, we evaluated its effect on A_2A_ and A_3_AR expression. The treatment of T/C-28a2 chondrocytes and hFOB 1.19 osteoblasts with IL-1β for 48 hours mediated a significant increase of A_2A_ and A_3_ARs (Table 2). Moreover, when T/C-28a2 and hFOB 1.19 cells were exposed to PEMFs for the last 24 hours during IL-1β treatment, we found a further increase of A_2A_ and A_3_AR density (Table 2).

**Table 2:** Affinity and density of A_2A_ and A_3_ARs in untreated or PEMF treated T/C-28a2 chondrocytes or hFOB 1.19 osteoblasts before and after IL-1β treatment.

	A_2A_ARs		A_3_ARs	
	K_D_	Bmax	K_D_	Bmax
**T/C-28a2**	2.72±0.23	126±10	3.04±0.28	131±12
**+PEMFs**	2.96±0.28	297±22*	3.21±0.27	288±25*
**+IL-1β**	2.83±0.23	325±31#	2.97±0.26	332±30#
**+PEMFs+IL-1β**	2.78±0.24	664±56* #	3.12±0.28	649±58* #
**hFOB 1.19**	2.52±0.21	110±11	3.02±0.26	135±12
**+PEMFs**	2.47±0.22	275±23*	3.21±0.27	328±29*
**+IL-1β**	2.45±0.22	349±34#	3.04±0.28	372±36#
**+PEMFs+IL-1β**	2.34±0.21	708±62* #	3.21±0.27	745±63* #

Affinity and density are expressed as K_D_, nM and Bmax, fmol/mg protein, respectively. Data are expressed as mean (n = 6) ± SEM.

* , p<0.01 vs the examined cells in the absence of PEMFs;

# , p<0.01 vs the examined cells in the absence of IL-1β.

### Immunofluorescence staining confirmed the PEMF-induced overexpression of A_2A_ and A_3_ARs in T/C-28a2 and hFOB 1.19 cells

**Figure 5:**
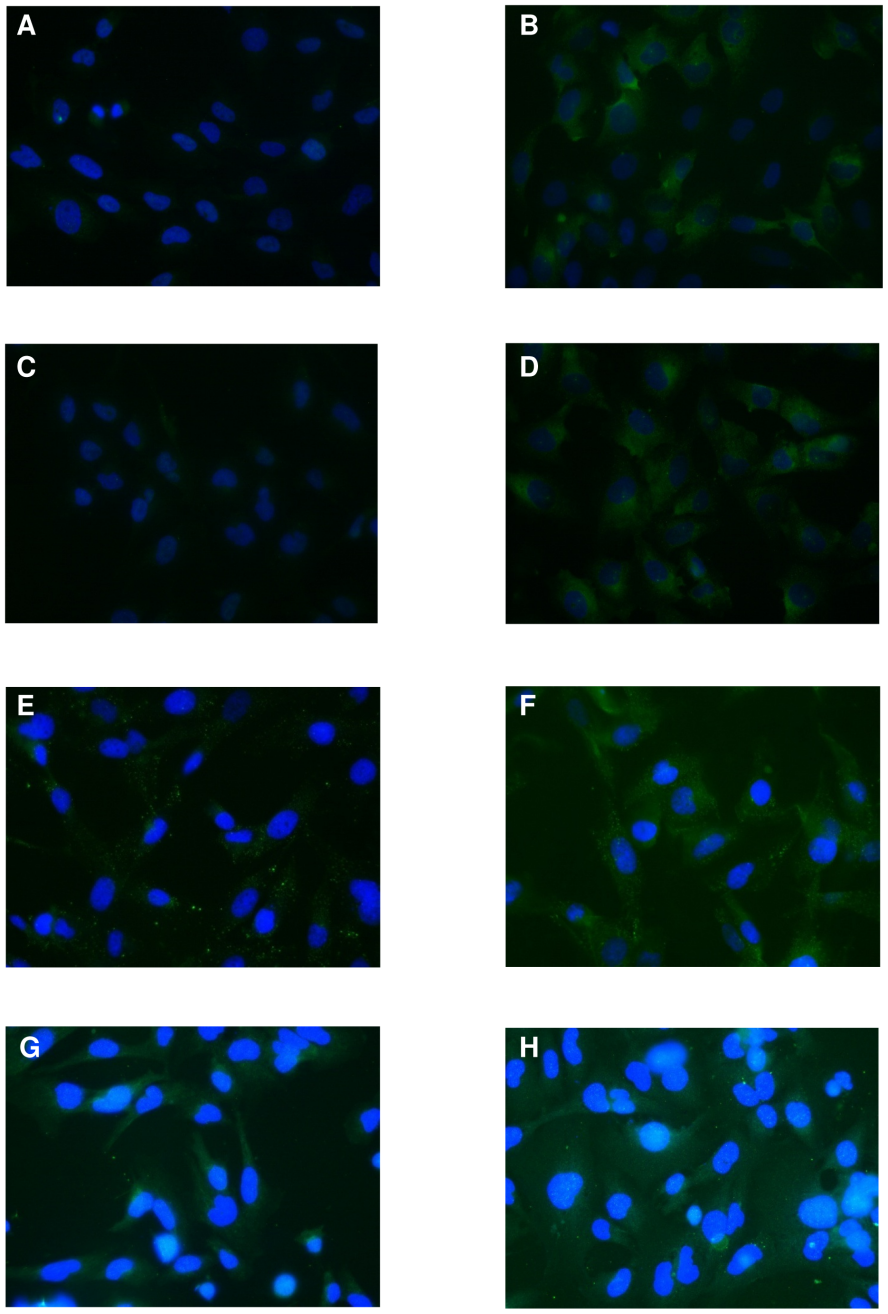
A_2A_ and A_3_AR immunofluorescence staining in T/C-28a2 and hFOB 1.19 cells. Effect of PEMF treatment on T/C-28a2 chondrocytes and hFOB 1.19 osteoblasts on A_2A_ and A_3_AR expression, determined by immunofluorescence experiments. DAPI was used for counterstaining of nuclei. A_2A_ (A, B) and A_3_AR (C, D) expression on T/C-28a2 chondrocytes in control condition (A, C) and after 24 hours exposure to PEMFs (B, D). A_2A_ (E, F) and A_3_AR (G, H) expression on hFOB 1.19 osteoblasts in control condition (E, G) and after 24 hours exposure to PEMFs (F, H). Original magnification 400×.

Immunoflourescence analysis demonstrate the presence of A_2A_ and A_3_ARs in T/C-28a2 chondrocytes (Figure 5, panel A and C, respectively). Interestingly, PEMF treatment determined an overexpression of these receptor subtypes as shown by the increase of the fluorescent signal (Figure 5B and D). The presence of A_2A_ and A_3_ARs in hFOB 1.19 was demonstrated by immunofluorescence analysis and, analogously to T/C-28a2 cells, PEMF exposure for 24 hours determined their upregulation (Figure 5E–H).

### PEMF exposure enhanced the differential effects of A_2A_ and A_3_AR stimulation on cAMP production and cell proliferation in T/C-28a2 and hFOB 1.19

**Figure 6:**
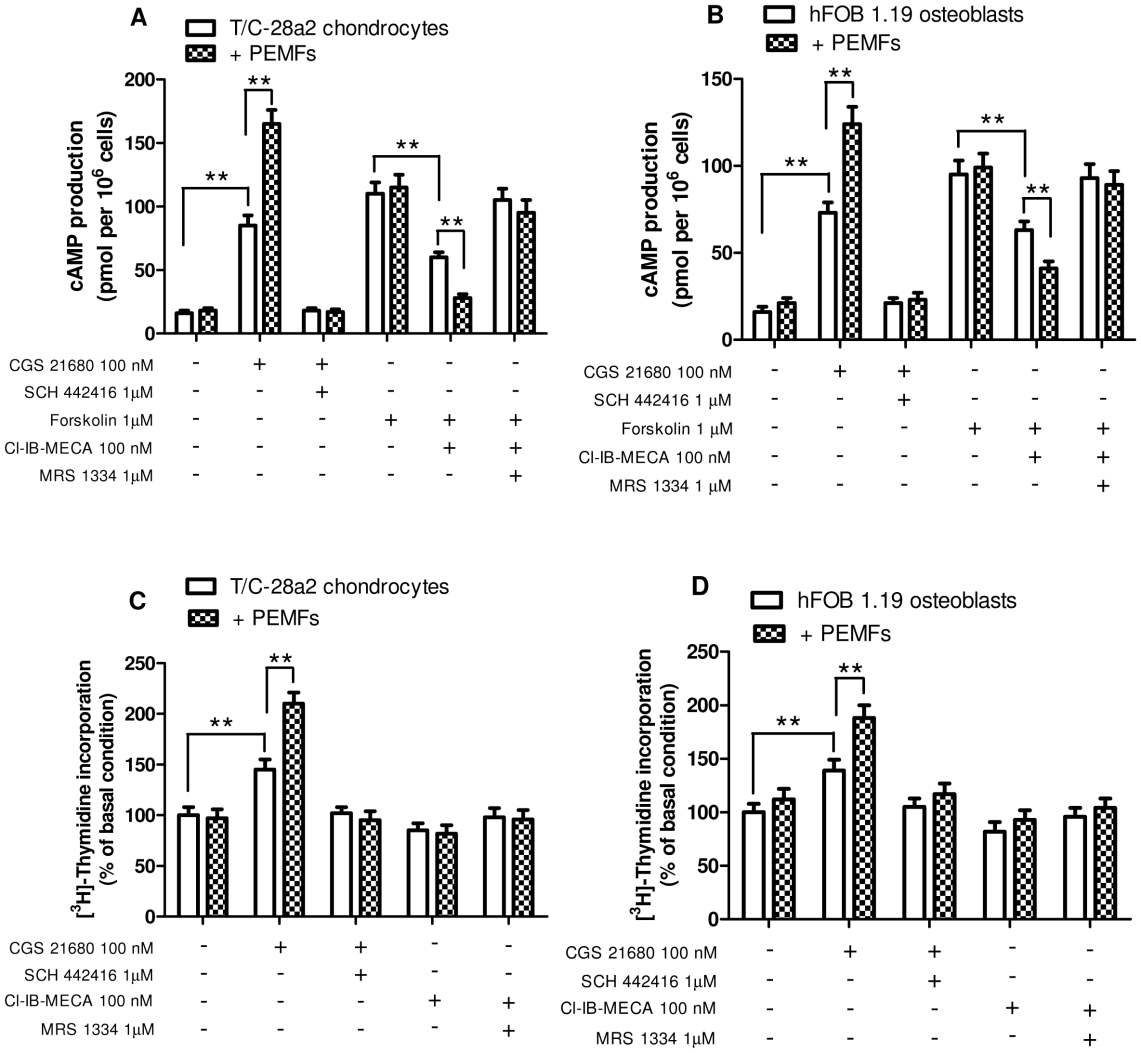
Modulation of cAMP production and cell proliferation in T/C-28a2 chondrocytes and hFOB 1.19 osteoblasts. Effect of CGS 21680 (100 nM) and Cl-IB-MECA (100 nM) on cAMP production in the absence or in the presence of SCH 442416 (1 µM) or MRS 1334 (1 µM) before and after PEMF exposure for 24 hours in T/C-28a2 chondrocytes (A) and in hFOB 1.19 osteoblasts (B). Forskolin (1 µM) was used to better evaluate the inhibitory effect of Cl-IB-MECA. Effect of A_2A_AR agonist and antagonist (CGS 21680, 100 nM; SCH 442416, 1 µM) or A_3_AR agonist and antagonist (Cl-IB-MECA, 100 nM; MRS 1334, 1 µM) on cell proliferation in the absence or in the presence of PEMF exposure for 24 hours in T/C-28a2 chondrocytes (C) and in hFOB 1.19 osteoblasts (D). Results are reported as the mean ± SEM of four independent experiments. *, p<0.05; **, p<0.01.

To evaluate if the upregulation of A_2A_ and A_3_ARs determined by PEMF in the examined cells was accompanied by an increase of their functional responses, we studied the cAMP production induced by A_2A_ and A_3_AR stimulation before and after 24 hours of PEMF exposure. As expected, in T/C-28a2 cells the well-known A_2A_AR agonist CGS 21680 (100 nM) elicited an increase of cAMP from a basal condition of 16±2 to 85±8 pmol/10^6^ cells. The treatment with PEMF enhanced the stimulatory effect of CGS 21680 that reached a cAMP production of 165±11 pmol/10^6^ cells (p<0.01 vs CGS 21680 in control conditions). The selective A_2A_AR antagonist SCH 442416 (1 µM) was able to abrogate the effect of CGS 21680 (Figure 6A). The inhibitory effect of the A_3_AR agonist Cl-IB-MECA was studied in the presence of the adenylate cyclase direct activator Forskolin (1 µM). While Cl-IB-MECA at the 100 nM concentration was able to inhibit the forskolin-stimulated cAMP levels by 45%, the pre-treatment of T/C-28a2 chondrocytes with PEMFs increased the effect of the A_3_AR agonist that was able to reduce the cAMP levels by 76% (p<0.01 vs Cl-IB-MECA in control conditions, Figure 6A). The A_3_AR antagonist MRS 1334 blocked the effect of Cl-IB-MECA in the presence or in the absence of PEMF exposure. Analogous results were obtained in hFOB 1.19 cells, suggesting the capability of PEMF exposure to increase the specific effects of A_2A_ or A_3_AR agonists on cAMP production (Figure 6B).

Chondrocyte and osteoblast cell proliferation represent an important issue in the cartilage and bone metabolism. For this reason we have evaluated the effect of A_2A_ and A_3_AR agonists on T/C-28a2 and hFOB 1.19 cell proliferation in the absence or in the presence of PEMF exposure. CGS 21680 (100 nM) was able to significantly increase cell proliferation in both the cell lines examined (Figure 6C and D). This effect was potentiated by PEMF exposure that determined a further increase of [^3^H]-Thymidine incorporation of 45% and 36% in T/C-28a2 and hFOB 1.19 cells, respectively (p<0.01 vs CGS 21680 in control conditions). The use of the selective A_2A_AR antagonist SCH 442416 (1 µM) that completely abrogated the CGS 21680-induced proliferation increase in the absence or in the presence of PEMFs, confirmed that this effect was determined by A_2A_AR activation. The A_3_AR agonist Cl-IB-MECA did not influenced the proliferation rate of T/C-28a2 chondrocytes or hFOB 1.19 osteoblast neither in the absence nor in the presence of PEMF exposure (Figure 6C and D).

### The anti-inflammatory effects of A_2A_ and A_3_AR activation are enhanced by PEMF exposure

**Figure 7:**
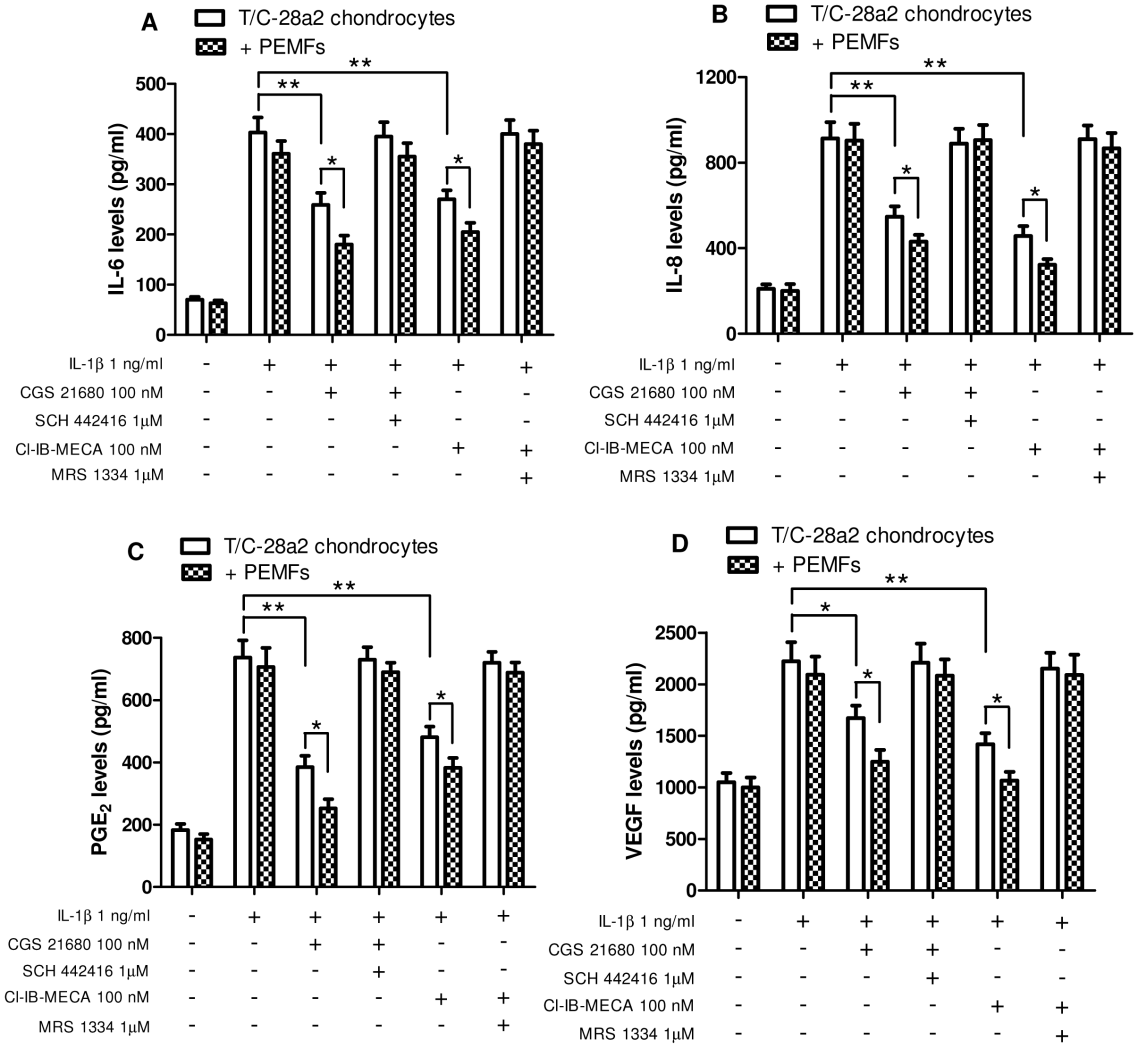
Inhibition of IL-6, IL-8, PGE_2_ and VEGF by A_2A_ or A_3_AR agonists and PEMFs in T/C-28a2 chondrocytes. Effect of A_2A_AR agonist and antagonist (CGS 21680, 100 nM; SCH 442416, 1 µM) or A_3_AR agonist and antagonist (Cl-IB-MECA, 100 nM; MRS 1334, 1 µM) on IL-6 (A), IL-8 (B), PGE_2_ (C) or VEGF (D) production in the absence or in the presence of PEMF exposure for 24 hours in T/C-28a2 chondrocytes stimulated with IL-1β (1 ng/ml). Results are reported as the mean ± SEM of four independent experiments. *, p<0.05; **, p<0.01.

The A_2A_ and A_3_AR agonists were both able to inhibit the IL-1β-stimulated release of pro-inflammatory cytokine IL-6 and IL-8 in T/C-28a2 and hFOB 1.19 cells. In particular, in T/C-28a2 chondrocytes the A_2A_AR agonist CGS 21680 (100 nM) elicited a reduction of IL-6 and IL-8 levels of 43% and 52%, respectively (Figure 7A and B). In the same cells, the A_3_AR agonist Cl-IB-MECA (100 nM) mediated an inhibition of IL-6 and IL-8 levels of 40% and 65%, respectively. Interestingly, the simultaneous treatment with A_2A_ and A_3_AR agonists and PEMFs resulted in a major effect on the inhibition of these pro-inflammatory cytokines. As expected, the use of selective A_2A_ and A_3_AR antagonists (SCH 442416 and MRS 1334, respectively) abrogated the effect of the agonists either in the absence or in the presence of PEMFs (Figure 7A and B).

In T/C-28a2 chondrocytes, the lipid mediator PGE_2_ levels stimulated by IL-1β were significantly reduced in the presence of CGS 21680 or Cl-IB-MECA and their inhibitory effects were potentiated by PEMF exposure that elicited a further reduction of 34% and 26%, respectively (p<0.05 vs CGS 21680 or Cl-IB-MECA in control condition, Figure 7C). We next investigated the release of VEGF in the same experimental conditions. PEMF exposure potentiated the inhibitory effect of A_2A_ and A_3_AR activation on IL-1β-stimulated VEGF production in T/C-28a2 cells (Figure 7D). SCH 442416 and MRS 1334 were able to counteract the responses mediated by A_2A_ and A_3_AR, respectively, suggesting the specific involvement of these receptor subtypes.

**Figure 8:**
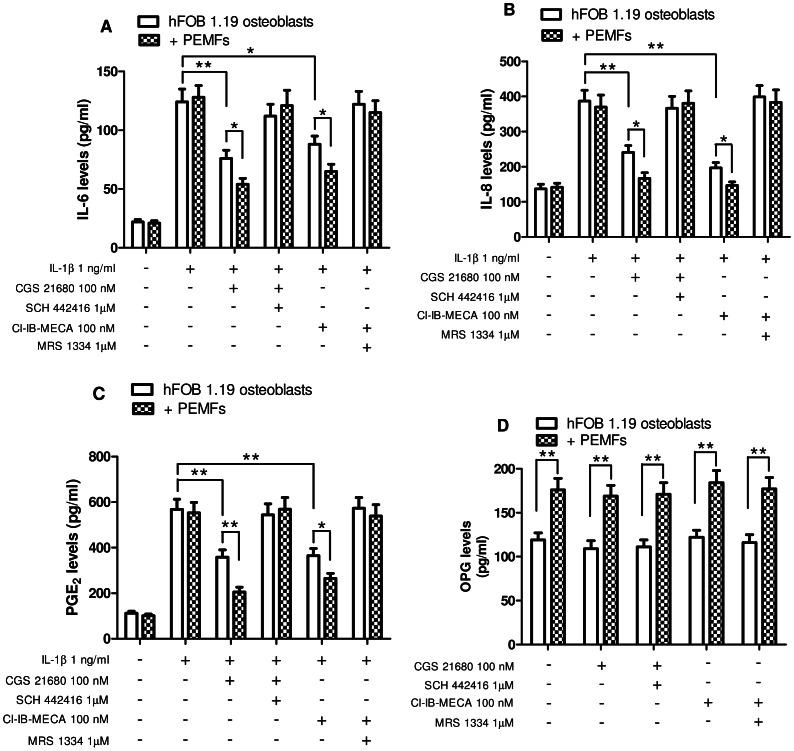
Modulation of IL-6, IL-8, PGE_2_ and OPG by A_2A_ or A_3_AR agonists and PEMFs in hFOB 1.19 osteoblasts. Effect of A_2A_AR agonist and antagonist (CGS 21680, 100 nM; SCH 442416, 1 µM) or A_3_AR agonist and antagonist (Cl-IB-MECA, 100 nM; MRS 1334, 1 µM) on IL-6 (A), IL-8 (B), PGE_2_ (C) or OPG (D) production in the absence or in the presence of PEMF exposure for 24 hours in T/C-28a2 chondrocytes stimulated with IL-1β (1 ng/ml). Results are reported as the mean ± SEM of four independent experiments. *, p<0.05; **, p<0.01.

IL-6, IL-8 and PGE_2_ release was also investigated in hFOB 1.19 osteoblasts obtaining similar results to those found in T/C-28a2 chondrocytes (Figure 8A, B and C). To investigate if the co-treatment with both A_2A_ and A_3_AR agonists determined an even further inhibition of IL-6 as an example of inflammatory mediator we incubated T/C-28a2 or hFOB 1.19 cells with CGS 21680 (100 nM) and Cl-IB-MECA (100 nM). The simultaneous treatment with the two agonists inhibited IL-1β-stimulated IL-6 release of 61% and 57% in T/C-28a2 and hFOB 1.19 cells, respectively, resulting in a higher inhibition than the single agonists.

Then, in hFOB 1.19 cells we have examined the effect of A_2A_ and A_3_AR agonists in the absence or in the presence of PEMFs on the production of OPG, a protein involved in bone metabolism. Neither CGS 21680 nor Cl-IB-MECA at the 100 nM concentration were able to modify the basal release of OPG. Interestingly, PEMF exposure significantly augmented the production of this protein eliciting an increase of 48% respect to basal conditions (p<0.01, Figure 8D). The release of osteoprotegerin following the treatment with CGS 21680 or Cl-IB-MECA in the presence of PEMFs was not different from that obtained with PEMF exposure alone suggesting an effect not related to the capability of PEMFs to modulate ARs signaling (Figure 8D).

### Effect of A_2A_ and A_3_AR agonists and PEMFs on NF-kB activation

**Figure 9:**
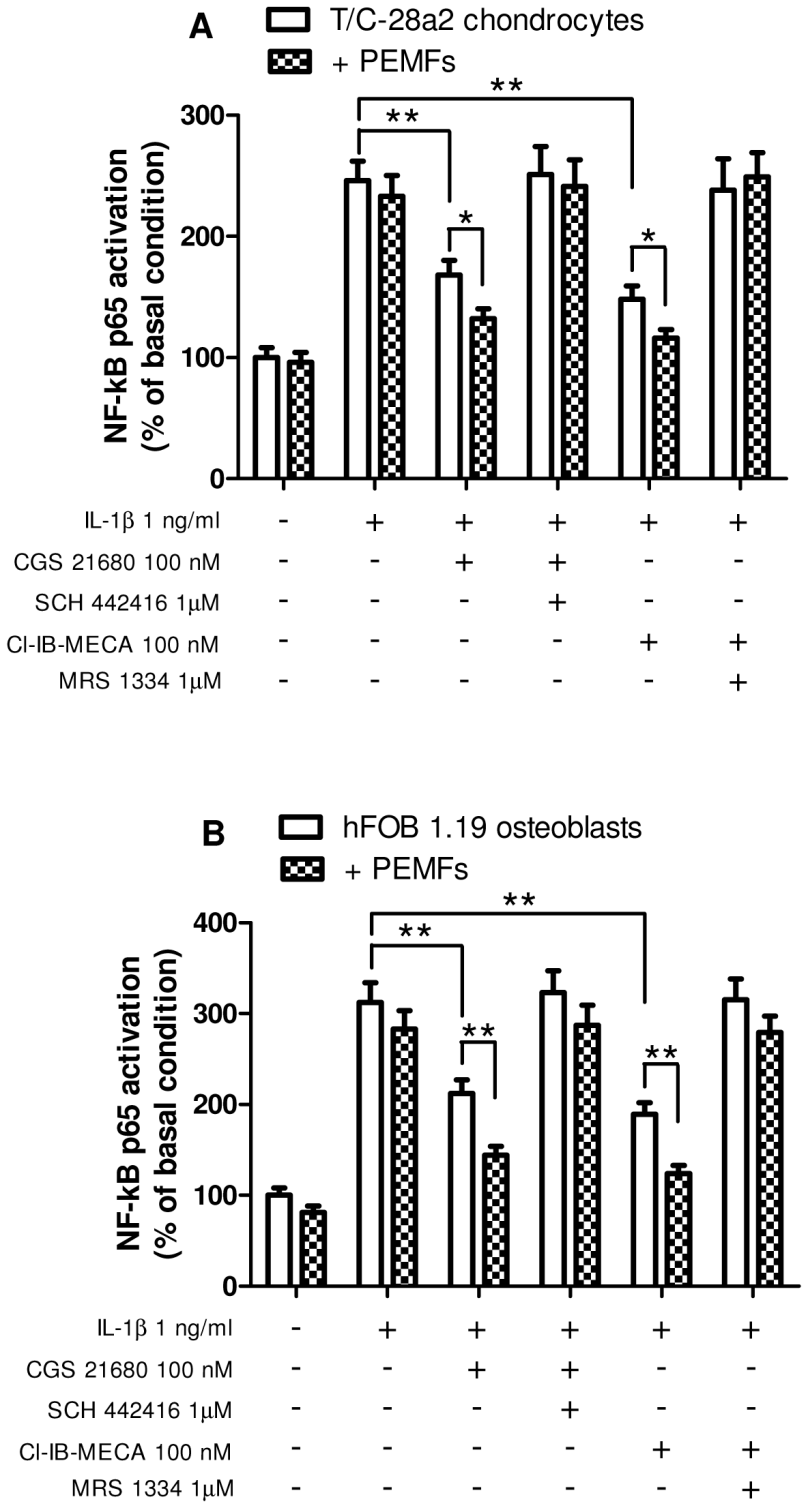
Inhibition of NF-kB activation by A_2A_ or A_3_AR agonists and PEMFs in T/C-28a2 and hFOB 1.19 cells. Effect of A_2A_AR agonist and antagonist (CGS 21680, 100 nM; SCH 442416, 1 µM) or A_3_AR agonist and antagonist (Cl-IB-MECA, 100 nM; MRS 1334, 1 µM) on NF-kB p65 subunit activation in the absence or in the presence of PEMF exposure for 24 hours in T/C-28a2 chondrocytes (A) or in hFOB 1.19 osteoblasts (B) stimulated with IL-1β (1 ng/ml). Results are reported as the mean ± SEM of four independent experiments. *, p<0.05; **, p<0.01.

The transcription factor NF-kB is a key regulator of inflammatory responses and plays a critical role also in cartilage and bone metabolism. For this reason we have evaluated the effect of CGS 21680 and Cl-IB-MECA in the absence or in the presence of PEMFs on NF-kB p65 subunit activation. Both A_2A_ and A_3_AR activation resulted in the inhibition of NF-kB stimulated with IL-1β in T/C-28a2 chondrocytes (Figure 9A) and hFOB 1.19 osteoblasts (Figure 9B). This effect was potentiated by the presence of PEMFs that were able to further inhibit the NF-kB p65 subunit activation. In both cell line, the use of the selective antagonists SCH 442416 and MRS 1334 counteracted the effect of the A_2A_ and A_3_AR agonists, respectively (Figure 9).

## Discussion

In this study we demonstrated that PEMF exposure mediates a specific overexpression of A_2A_ and A_3_AR in T/C-28a2 chondrocytes and hFOB 1.19 osteoblasts. This effect was confirmed both at a transcriptional level, as shown by RT-PCR assays, as well as in the increase of protein expression, as revealed by Western blotting, immunofluorescence and saturation binding experiments. On the other hand, PEMF treatment did not affected A_1_ and A_2B_AR mRNA or protein expression. These data are consistent with those previously reported by our group showing that PEMF exposure are able to induce the overexpression of A_2A_ and A_3_ARs in different cell types such as human neutrophils [28], [29], bovine chondrocytes and synoviocytes [32] and neural cancer cells [31]. Given the well-recognized anti-inflammatory effects of A_2A_ and A_3_AR activation, the increase of their density following IL-1β stimulation could be interpreted as a compensatory mechanism to counteract excessive inflammation. This is in agreement with data previously reported showing the up-regulation of A_2A_ and A_3_ARs in cells or tissues from patients affected by different inflammation-based pathologies such as chronic obstructive pulmonary disease and rheumatoid arthritis [20], [21], [23]. The further increase of A_2A_ and A_3_AR density elicited by PEMFs could indicate a potentiation of this compensatory mechanism, suggesting the possibility to exploit the PEMF-induced A_2A_ and A_3_AR upregulation to reduce the inflammatory status. Taken together, these results suggest that at least some of the effect elicited by PEMFs in biological systems could be attributed to the modulation of these AR subtypes. The mechanism by which PEMFs determined an up-regulation of A_2A_ and A_3_ARs is not yet understood. Several evidence report that PEMF could act either at a membrane level or at a transcriptional level [15], [16], [28]–[33]. Nevertheless, further studied are necessary to elucidate the exact mode of action of PEMFs. To verify if the PEMF-induced up-regulation of A_2A_ and A_3_ARs was accompanied by altered receptor functional responses, we have performed cAMP experiments before and after PEMF treatment. The capability of PEMFs to potentiate the typical responses of A_2A_ and A_3_AR agonists on cAMP production suggested the synergistic use of biophysical stimulation to enhance the well-known anti-inflammatory effect of A_2A_ and A_3_AR activation.

Articular cartilage may undergo repeated damage involving a degenerative process that includes focal and progressive cartilage loss [45]. Moreover, clinical observation has shown that trauma, cancer, osteoporosis and osteoarthritis can lead to loss of mechanical bone competence and to bone resorption [46]. The main issue in the treatment of these diseases is how to increase chondrocyte proliferation to promote cartilage repair or how to generate more osteoblasts to promote ossification and accelerate osteogenesis. Our data showed that the A_2A_AR agonist CGS 21680 had a positive effect on both chondrocytes and osteoblasts proliferation, and the simultaneous presence of PEMF exposure potentiated this proliferative action. Thus, the two agents combined could represent a potential alternative strategy for the treatment of pathological conditions characterized by an excessive cartilage degradation or bone resorption. Previous reports in the literature have documented that PEMF exposure may have a proliferative effect on both chondrocytes and osteoblast, although after a longer culture period [47]–[49]. This may suggest that the presence of the A_2A_AR agonist could accelerate this process. On the other hand, the A_3_AR agonist Cl-IB-MECA did not affected cell proliferation nor in the presence or in the absence of PEMF exposure.

**Figure 10:**
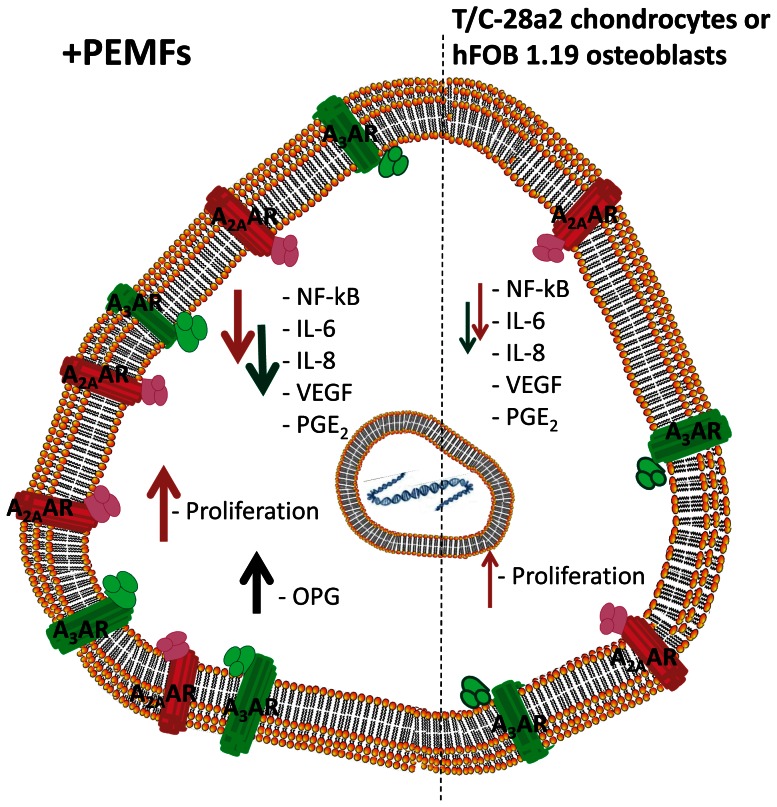
Proposed mechanism of anti-inflammatory effect of PEMFs through the up-regulation of A_2A_ and A_3_ARs in T/C-28a2 and hFOB 1.19 cells. PEMF exposure induce an up-regulation of A_2A_ and A_3_ARs in T/C-28a2 chondrocytes and hFOB 1.19 osteoblast. As a consequence, the anti-inflammatory effects of A_2A_ (red arrows) and A_3_AR (green arrows) activation (on the right) are enhanced in the presence of PEMF exposure (on the left) resulting in a further inhibition of pro-inflammatory cytokines, NF-kB, VEGF, PGE_2_. PEMFs increased the effect of A_2A_AR stimulation on cell proliferation in both cell lines and determined a marked production of OPG in hFOB 1.19 osteoblasts (black arrow).

Inflammatory mediators play crucial roles in cartilage degenerative conditions as well as in bone metabolism. In this study, we have evaluated the effect of A_2A_ and A_3_AR stimulation in the absence or in the presence of PEMFs on pro-inflammatory cytokine release from T/C-28a2 chondrocytes and hFOB 1.19 osteoblasts. The release of inflammatory mediators were stimulated by using Il-1β, a pro-inflammatory cytokines that interacts with most cell type and is one of the most important mediator of the inflammatory response, especially in cartilage and bone pathologies. In both the cell line examined, CGS 21680 and Cl-IB-MECA were able to significantly decrease the IL-1β-stimulated production of the inflammatory mediators IL-6, IL-8 and PGE_2_. In T/C-28a2, a similar effect was observed on the production of VEGF, an important mediator of angiogenesis. The data are consistent with those previously found in chondrocytes and/or osteoblasts. In particular, it has been recently shown that A_2A_AR activation reduced inflammatory mediators in mouse articular chondrocytes stimulated with IL-1β [50]. Moreover, in the osteoblastic cell line MG-63, the treatment with adenosine inhibited IL-6 production via A_2A_AR activation [51]. The capability of both A_2A_ and A_3_AR agonists to mediate anti-inflammatory effect suggests the involvement of different downstream signaling pathways. As a matter of fact, in a previous work we demonstrated that the anti-inflammatory effects of A_2A_ARs were mediated by the modulation of cAMP. In contrast, the inhibitory effect of A_3_AR activation on pro-inflammatory mediators was completely abrogated by using the PI3K inhibitor LY294002 but not by the Gi inactivator pertussis toxin [40]. Interestingly, the present study highlighted the capability of PEMF exposure to potentiate the anti-inflammatory effects mediated by A_2A_ and A_3_AR agonists. Numerous evidence have suggested that PEMFs possess a potential anti-inflammatory effect [15]–[17], [52] and our new data indicate that the up-regulation of A_2A_ and A_3_ARs could be considered one of the mechanism by which PEMFs exerted their effects (Figure 10).

Osteoprotegerin is a competitive protein for receptor activator of nuclear factor kappa-B ligand (RANKL) and has been shown to prevent bone resorption by blocking the binding of RANKL with the receptor RANK, thereby inhibiting osteoclast differentiation and activation [53]. Our results obtained from hFOB 1.19 osteoblasts revealed that, although A_2A_ and A_3_AR stimulation had no effect on osteoprotegerin production, PEMF exposure resulted in a significant increased release of this bone protective factor. These data are consistent with various papers previously reported where PEMF exposure increased osteoprotegerin secretion and mRNA expression in osteoblast-like cells [54], [55]. Moreover, it has been shown that PEMFs stimulated osteoprotegerin in rats preventing ovariectomy-induced bone loss [56].

The activation of the transcription factor NF-kB is known to be central for the regulation of the synthesis and activity of inflammatory cytokines, including TNF-α and IL-1β, and also several other mediators involved in the pathogenesis of inflammatory joint and bone diseases [57]. In addition, NF-kB controls the differentiation or activity of the major skeletal cell types such as osteoclasts, osteoblasts, osteocytes and chondrocytes [58]. It is well-known that many of the anti-inflammatory effects of A_2A_ and A_3_AR stimulation are mediated by the inhibition of NF-kB signaling pathway [59], [60]. In the present study, we provide evidence that A_2A_ and A_3_AR agonists were able to reduce the IL-1β-induced NF-kB p65 subunit activation also in T/C-28a2 chondrocytes and hFOB 1.19 osteoblasts. Interestingly, the simultaneous exposure of the cells with PEMFs enhanced this inhibitory effect, suggesting the potential utilization of PEMFs and A_2A_ and A_3_AR agonists to reduce overactivation of NF-kB.

In conclusion, our data revealed that PEMFs mediated an up-regulation of A_2A_ and A_3_ARs in T/C-28a2 chondrocytes and hFOB 1.19 osteoblasts. The PEMF-induced overexpression is accompanied by a potentiation of the typical anti-inflammatory responses elicited by A_2A_ and A_3_AR activation. In addition, PEMFs regulated the production of the bone-protective factor osteoprotegerin from hFOB 1.19 cells. Taken together, these results suggest the potential for a combined use of two different approaches represented by pharmacological tools such as A_2A_ and A_3_AR agonists and a biophysical stimuli such as PEMFs in order to modulate cartilage and bone activity, especially in inflammatory conditions.
